# Multiplexed single-molecule force spectroscopy using a centrifuge

**DOI:** 10.1038/ncomms11026

**Published:** 2016-03-17

**Authors:** Darren Yang, Andrew Ward, Ken Halvorsen, Wesley P. Wong

**Affiliations:** 1 School of engineering and applied sciences, Harvard University, Cambridge, Massachusetts 02138, USA; 2 Program in Cellular and Molecular Medicine, Boston Children's Hospital, Boston, Massachusetts 02115, USA; 3 Department of Biological Chemistry and Molecular Pharmacology, Harvard Medical School, Boston, Massachusetts 02115, USA; 4 The RNA Institute, University at Albany, Albany, New York 12222, USA; 5 Wyss Institute for Biologically Inspired Engineering, Harvard University, Boston, Massachusetts 02115, USA

## Abstract

We present a miniature centrifuge force microscope (CFM) that repurposes a benchtop centrifuge for high-throughput single-molecule experiments with high-resolution particle tracking, a large force range, temperature control and simple push-button operation. Incorporating DNA nanoswitches to enable repeated interrogation by force of single molecular pairs, we demonstrate increased throughput, reliability and the ability to characterize population heterogeneity. We perform spatiotemporally multiplexed experiments to collect 1,863 bond rupture statistics from 538 traceable molecular pairs in a single experiment, and show that 2 populations of DNA zippers can be distinguished using per-molecule statistics to reduce noise.

## Abstract (web summary)

Single molecule force spectroscopy (SMFS) has limitations in throughput and the ability to repeatedly interrogate single bonds. Here the authors repurpose a benchtop centrifuge and use DNA nanoswitches to enable high throughput SMFS capable of repeatedly measuring forces of single molecular pairs.

## Graphical abstract

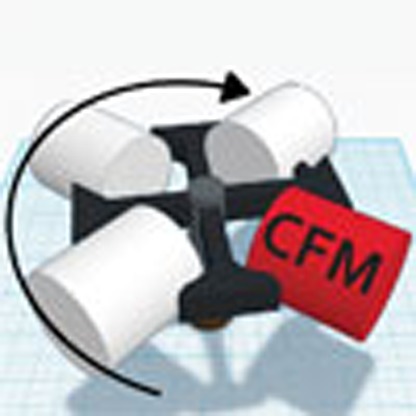

The ability to mechanically manipulate single molecules is leading to insights throughout biomedical research, from the action of molecular motors in replication and transcription to the role of mechanical forces in development^1^,^2^,^3^,^4^,^5^. While in principle these approaches enable the full characterization of individual molecular complexes and the study of population heterogeneity at the single-molecule level, in practice key challenges exist. The first challenge for force spectroscopy studies is the low throughput of most single-molecule approaches, which is just starting to be addressed with recently developed multiplexed methods^6^,^7^,^8^,^9^,^10^,^11^,^12^,^13^. Furthermore, sufficient statistics must be collected not only for the population^14^, but also for each individual molecule, which can be a challenge for studying catastrophic transitions such as bond rupture^15^,^16^,^17^. Another challenge is the positive identification of the single-molecule interactions of interest over non-specific and multiple interactions. Finally, there is the subtle challenge of noise, both thermal and experimental, that makes distinguishing different populations of molecules with similar force properties difficult.

We have met all of these challenges with spatiotemporally multiplexed force spectroscopy, a combination of massively parallel spatial multiplexing with repeated interrogation enabled by self-assembled nanoscale devices. First, we introduce a miniature centrifuge force microscope (CFM) for high-throughput single-molecule experimentation that utilizes a commercial benchtop centrifuge. Functionally similar to our first prototype device^18^, an entire microscope imaging system is rotated to observe microscopic objects subjected to uniform centrifugal force (unlike earlier ‘spinning disk' centrifuge microscopes^19^,^20^). This inexpensive design brings new features including temperature control and high-resolution particle tracking (∼2 nm). Second, we introduce a high-throughput CFM assay that integrates mechanical nanoswitches^17^,^21^,^22^ to provide important new functionality. The nanoswitches serve two roles—one as a molecular signature to facilitate reliable and automated analysis of large data sets, and the second to enable the repeated interrogation of each single-molecule pair, increasing throughput and enabling new measurements of heterogeneity in single-molecule experiments. By making repeated force measurements on hundreds of single-molecule complexes, we can collect multiple statistics on each molecule that comprises the population. We additionally show that by averaging multiple rupture forces on a per-molecule basis, we can reduce noise to enable super-resolved force spectroscopy—the identification of different populations of molecules below the thermal force-resolution limit. Averaging allows us to reduce the spread in force distributions (averaging reduces noise by a factor of ∼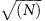) without losing information about differences between molecules. Furthermore, the rich and relatively large data sets provided by our technique could also complement other analysis techniques for statistical deconvolution^23^,^24^.

## Results

### Benchtop CFM with nanoscale tracking

**Figure 1:**
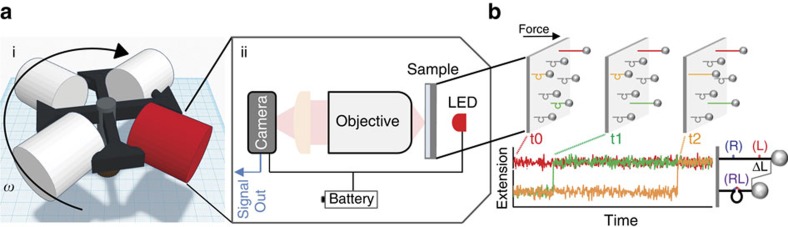
Overview of the benchtop CFM. (**a**,i) Illustration of a spinning centrifuge rotor containing the CFM in the red swing bucket; the rotation induces forces on molecules tethered between beads and the coverslip surface. The direction of force application can be set by constraining the angle of the centrifuge bucket, as described in Supplementary Fig. 5. (**a**,ii) Schematic of the CFM. (**b**) Tether extension is monitored as a function of time while force is applied. When the DNA nanoswitch is integrated, molecular transitions such as bond rupture between a receptor (R)–ligand (L) pair causes a well-defined change in tether extension, providing a distinct signature for detecting interactions between two molecules of interest.

The redesigned CFM is a miniaturized microscope that fits into a commercial centrifuge so that force can be applied to samples while their micro- to nano-scale motions are tracked (Fig. 1 and Supplementary Fig. 1). To accommodate the microscope components in the 400 ml bucket volume, we selected components with size and weight in mind, and induced two right-angle bends in the optical path (Supplementary Fig. 1b). To ensure accessibility of the instrument, most components are commercially available and can be easily assembled (Supplementary Fig. 1d,e and Supplementary Table 1). A three-dimensional (3D)-printed housing was made to encapsulate the device and to ensure a tight fit within the bucket (Solidworks drawings provided as Supplemental Files). Transmission of camera data out of the CFM during centrifugation is accomplished by converting the camera's gigabit Ethernet signal to a fibre-optic signal, then passing this data out of the centrifuge through a fibre rotary joint (Supplementary Fig. 1a,f). Details of the design are presented in the Methods.

We implemented a unique solution to measure nanometer-level extensions of tethers in the CFM by projecting tether length changes onto the *x–y* plane of the coverslip, enabling these measurements to be made in a relatively simple and computationally efficient way. Taking advantage of the fact that we can precisely control the direction of force application by mechanically constraining the angle of the centrifuge bucket, we intentionally misaligned the force and imaging axes (Supplementary Fig. 2a and Supplementary Note 1). Using this technique, we can ‘tune' our tether length resolution based on the bucket angle with a range of ∼2.5 nm (at 20°) to 12 nm (at 80°) based on our lateral tracking resolution of ∼2 nm (Supplementary Fig. 2b,c). To validate and demonstrate this approach, we measured DNA force extension for over 100 molecules simultaneously over a span of <1 min (Supplementary Fig. 3a), and fit each with the standard worm-like chain model. The most likely contour length and persistence length was 8.2±0.2 μm and 46±1 nm, respectively, in agreement with expected values^25^. We additionally performed multiplexed overstretching measurements of lambda DNA (Supplementary Fig. 3c), yielding an overstretching force of 63.5±1.7 pN (mean±s.d.), consistent with previous measurements at these conditions^25^.

### DNA nanoswitches for authenticating single-molecule data

**Figure 2:**
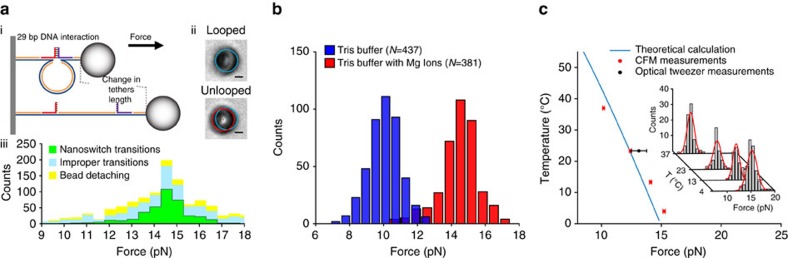
DNA unzipping force measured using DNA nanoswitches on the CFM. (**a**,i) Schematic of the unzipping construct. Two complementary oligos (red and purple strands) hybridize to form a looped DNA nanoswitch. Force can unzip the two complementary strands, resulting in a measurable increase in tether length, providing a signature of DNA unzipping. (**a**,ii) Images of a bead tethered to the surface via a DNA nanoswitch showing the looped and unlooped states. The scale bar, 1-μm long. (**a**,iii) In one example of rupture-force measurement, we identified 381 tethers with the DNA nanoswitch transitions signature to collect rupture forces while the remaining 673 transitions that corresponded to bead detachment and improper transitions were omitted (see also Supplementary Fig. 5 for detail). (**b**) Unzipping force histograms of 29 bp dsDNA measured with the DNA nanoswitch under two different buffer conditions. (**c**) Average unzipping force of 29 bp dsDNA under different temperatures with PBS buffer (Total *n*=306), with histograms of rupture forces shown as an inset. The theoretical line is calculated using a previously described thermodynamic model^30^.

To enable robust and repeatable rupture experiments, we integrated DNA nanoswitches into the CFM. These molecular switches are designed to adopt a looped structure when the molecules of interest are interacting and a linear structure when they are not (Fig. 2a), providing a distinct ‘signature' (that is, increase in tether length) for rupture events (Supplementary Fig. 4b). We performed DNA unzipping experiments on a 29 bp DNA interaction, and used the ‘signature' unlooping of the nanoswitches to positively identify and discriminate valid single-molecule data from multiple tethers and non-specific interactions (Fig. 2a and Supplementary Fig. 5). Following this approach, unzipping force measurements were carried out in the presence and absence of magnesium ions, with hundreds of rupture statistics for each condition collected in under 30 s of centrifuge run time. We found that magnesium stabilizes the duplex as observed previously^26^, with the average unzipping force (±s.d.) increasing from 10.1±0.9 to 14.6±1.1 pN with the addition of magnesium (Fig. 2b).

### Temperature-dependent DNA unzipping

We additionally performed DNA unzipping experiments at four temperatures, 4, 13, 23 and 37 °C (Fig. 2c). Seminal works introduced temperature control into optical tweezers over 10 years ago^27^,^28^, but even today the vast majority of single-molecule pulling experiments are carried out at room temperature, and temperature control remains an active area of development^29^. The benchtop CFM has the advantages of built-in temperature control and portability to move into cold (4 °C) or warm (37 °C) rooms available to most biologists. Accurate, real-time measurements of the sample temperature during experiments were made with a wireless thermocouple embedded within the centrifuge bucket (Methods for full details). We found an increase in the unzipping force with decreasing temperature, in good quantitative agreement with the thermodynamic models of DNA unzipping and previous observations^30^ (see also Supplementary Note 2).

### Repeated interrogation and super-resolved force spectroscopy

**Figure 3:**
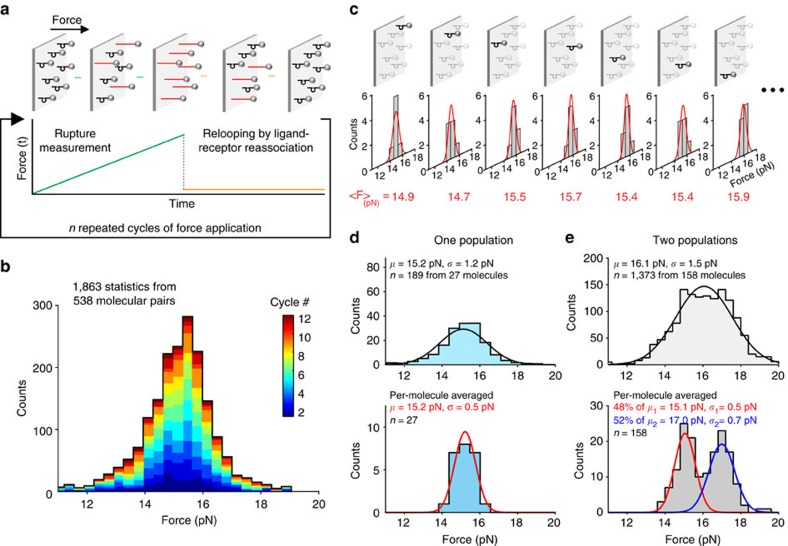
Repeated rupture-force measurement of single molecular pairs. (**a**) Protocol for repeated cycles of force application, with each cycle consisting of a linear force ramp to induce rupture and DNA nanoswitch unlooping, followed by a low force reassociation period allowing the molecular pairs to rebind. (**b**) DNA unzipping force histogram of 1,863 rupture events collected from a total of 538 molecular pairs with 12 cycles of force application. The colour from blue to red corresponds to statistics collected from each cycle. (**c**) Example rupture-force histograms generated for individual molecular pairs. The calculated average rupture force is shown below in red. (**d**) Combined histogram of rupture forces from 27 molecules with 7 cycles of force rupture each (top), and histogram of the per-molecule averaged rupture force (bottom), showing a reduced width. (**e**) Combined histogram for two populations of DNA unzipping experiments (top), and the per-molecule averaged super-resolved histogram (bottom) that recovers the two separate populations from the mixed data.

Finally, we demonstrate spatiotemporally multiplexed force spectroscopy using the DNA nanoswitch assay on the CFM to repeatedly interrogate a population of molecules at the single-molecule level (Fig. 3a). We measured 1,863 statistics of DNA unzipping from repeated pulls of 538 molecules in a single sample to demonstrate the large amounts of single-molecule force data that can be accumulated with this approach (Fig. 3b). Even more significantly, the nanoswitches enable the unique properties of each molecule in a sample to be characterized from repeated measurements, as we demonstrate by determining a rupture-force histogram for each molecule in a sample, illuminating population heterogeneity at the single-molecule level (Fig. 3c). Furthermore, we show that by averaging data from multiple pulls of the same molecular pair the spread in force is reduced without losing the unique characteristics of each molecule. When applied to data from a single population, this per-molecule force averaging generates a super-resolved histogram with the expected narrowing when compared with the raw histogram of all the data (Fig. 3d). When applied to combined statistics from two populations of DNA zippers (introducing another G–C-rich zipper with a higher unzipping force^31^), we show that the super-resolved histogram generated from per-molecule averaging can separate out two populations that are unresolveable from the raw histograms due to the intrinsic broadening of force that results from thermal noise and instrumental noise (Fig. 3e).

## Discussion

As we have shown, together the CFM and the DNA nanoswitch assay provide powerful single-molecule capabilities, and give non-specialists unprecedented access to force spectroscopy. And even separately these technologies provide unique benefits that can make single-molecule experiments better, cheaper and faster. Our miniature CFM presented here, which integrates into standard benchtop centrifuges, has important new features including an expanded biologically relevant dynamic force range (Supplementary Fig. 6), easily implemented temperature control between 4 and 37 °C, improved safety, lower cost (Supplementary Table 1 and Supplementary Note 3), and greater ease of use compared with the first-generation CFM. These last two points are important strides towards increasing the accessibility of single-molecule experiments for non-specialists. The total reduction in cost is particularly significant when considering the additional infrastructure typically needed for single-molecule measurements that are obviated by our approach, as the centrifuge itself provides an isolated, temperature-regulated enclosure. In addition, our instrument can be operated without significant training, even by undergraduate researchers. Similarly, the DNA nanoswitch-based single-molecule assay has its own benefits, significantly reducing the effort to obtain reliable and accurate measurements by standardizing sample preparation, and by supplying a distinct molecular signature that enables reliable data verification and automated analysis. Furthermore, it enables repeated interrogation of each molecular complex for building up statistics of both the population and the individual molecules, revealing population heterogeneity at the single-molecule level.

There is difficulty in generalized comparisons of single-molecule instruments due to the wide variety of designs, but the miniature CFM presented here (using the 2.8 μm dynabeads) is comparable to our own optical tweezers set-up^17^,^32^ in force range (∼0.1 to 100 pN), force resolution (∼3–5%) and spatial resolution (∼2 nm). It is worth noting, however, that the most advanced optical tweezers push spatial resolution into the sub-Angstrom range, have force resolution of <0.1 pN and have temporal resolutions >10 kHz (this CFM is 15 Hz)^2^. Regarding multiplexing, magnetic tweezers have achieved up to 357 statistics in a single experiment^8^, acoustic manipulation has achieved 145 statistics^6^ and other multiplexing methods show great promise as well^12^,^13^. However, our demonstration of 1,863 validated single-molecule statistics in one experiment is to our knowledge more than in any previous work.

As the potential for single-molecule approaches to address critical problems in biology becomes increasingly clear, the availability of such simple and powerful tools that do not require months of training or hundreds of thousands of dollars becomes a key. We believe the technologies presented here are an important step in that direction, enabling both a more detailed view of single molecules as part of a population, and a more streamlined way to amass large numbers of statistics. It is not unreasonable to imagine that this approach, paired with sample scanning and/or fast force cycling could eventually enable the collection of millions of single-molecule statistics from a single sample. With these advances, previously unrealistic applications such as high-throughput single-molecule screening and diagnostics become more viable, and single-molecule analysis may start to become as ubiquitous as standard bulk assays.

## Methods

### CFM instrumentation

The CFM consists of an optical microscope and digital image acquisition system, which is integrated into a centrifuge (Fig. 1a and Supplementary Fig. 1). We used a refrigerated benchtop centrifuge (Heraeus X1R, Thermo Scientific) with two modifications. First, the TX-400 rotor was modified to mount a fibre-optic rotary joint along the central axis (Princetel MJX). This was accomplished by removing the rotor's central push-release mechanism (by loosening the screw on the side of the button), and threading the four existing holes to accept 10–32 screws. An adapter was designed and installed on the rotor to hold the slip ring (Supplementary Fig. 1a,f). To enable the fibre optic to pass through the lid, the central plastic viewing window was removed. Second, the centrifuge's control module was upgraded to enable computer control. An upgraded module was kindly provided by Thermo Fisher Scientific to support this project, and was a simple drop-in replacement.

The light microscope of the benchtop CFM was constructed using mainly Thorlabs SM1 compatible components (Supplementary Fig. 1b,d,e and Supplementary Table 1). A red LED (LED630E, Thorlabs) threaded to a tube mount (S1LEDM, Thorlabs) served as the illumination source. A glass diffuser positioned between the LED source and sample cell provided uniform illumination across the field of view. The 25 mm diameter of the sample cell was designed to be compatible with the SM1 lens tube (see below CFM sample cell). The sample was magnified and imaged onto a CCD camera (GC 2,450, Prosilica, AVT) with a 40 × Olympus Plan Achromat objective (infinity corrected, 0.65 numerical aperture and 0.6 mm working distance) and Ø1″ 100 mm tube lens (AC254-100-A, Thorlabs). Due to the limited depth of the centrifuge TX-400 bucket (∼16 cm), we shortened the length of the microscope by bending the light path 180° using a pair of turning mirrors glued to a custom turning cube (part #17 in Supplementary Fig. 1e). Moreover, a 3D-printed enclosure made of acrylonitrile butadiene styrene was used to secure and integrate the imaging and acquisition system inside of the centrifuge bucket. The enclosure also included an open slot for a battery (PRT-00339, SparkFun), and a connected direct current (DC)-to-DC step-up circuit (PRT-08290, SparkFun) that served as the power source for the LED, camera and media converter. The camera used the standard GigE Vision interface, outputting the data as a gigabit Ethernet signal. To enable live imaging, we utilized a fibre-optic rotary joint installed at the centre of the centrifuge rotor (Supplementary Fig. 1f). The camera signal was converted from twisted-pair Ethernet to a fibre-optic signal by a small media converter inside of the centrifuge, transferred through the rotary joint, then converted back to a standard Ethernet signal by a second media converter connected to the acquisition computer. (855–10,734 and 855–10,735, IMC Networks) (Supplementary Fig. 1a). The images collected from the camera were recorded with custom LabView software developed with the Vision Module.

We embedded a portable wireless thermocouple connector (MWTC-D-K-915, Omega Engineering) with a surface adhesive thermocouple (SA1XL-K, Omega Engineering) within the bucket that contains the CFM to measure the sample temperature. A wireless receiver (WTC-REC1-915, Omega Engineering) was used to acquire the temperature from the thermocouple connector to record the temperature in real time.

Software and technical drawings are available as Supplemental Files and on request.

### Molecular constructs

The looped DNA nanoswitch construct was made using our previously published DNA self-assembly protocol^17^,^21^. Circular M13mp18 single-stranded DNA (ssDNA) (N4040S, New England Biolabs) was linearized by hybridizing a 40 bp oligo that created a double-stranded restriction site for the BtsCI enzyme (R0647S, New England Biolabs). Subsequently, a set of complementary oligos (Integrated DNA Technologies) was hybridized onto the linear ssDNA. Functionalized oligos (biotinylated and digoxigenin-modified) were hybridized onto the 3′ and 5′ ends of the ssDNA, respectively. The hybridization was carried out with 15 nM of linearized ssDNA and 10 M excess of the complementary oligos in 1 × NEBuffer 2 with a temperature ramp from 90 to 20 °C (−1 °C min^−1^) in a thermocycler. After this initial hybridization two specific single-stranded regions remained, which were bridged by two partially complimentary oligos to form the final looped construct (Fig. 2a). The sequence of the complimentary bridge oligo that formed the loop was: 3′-CTCAAATATCAAACCCTCAATCAATATCT-5′. This secondary hybridization step was carried out at a final construct concentration of 250 pM with a 1.25 molar excess of the bridge oligos in 1 × NE buffer 2 at room temperature for 1 h. We verified looping of the construct using gel-shift assays, single-molecule optical trap measurements and atomic force microscopy imaging (Supplementary Fig. 4). In the optical trap measurement, we applied force on the looped DNA construct via tethering between laser-trapped streptavidin and anti-digoxigenin-functionalized silica beads.

For the DNA overstretching measurements in the CFM, we functionalized both ends of lambda DNA with biotin to provide strong anchorage to the streptavidin-functionalized glass coverslip and bead surfaces. First, 20 μl of lambda DNA (0.28 μg ml^−1^, 10745782001, Roche) was incubated for 20 min at 65 °C to remove the hybridized overhangs. Subsequently a nucleotide mixture that consists of Biotin-14-dATP, Biotin-14-dCTP, dTTP and dGTP, each at 100 μM final concentration, was added to the lambda DNA solution with 0.25 U ml^−1^ Klenow Fragment (M0212S, New England Biolabs). This mixture was incubated for 1 h at 37 °C. The dual-end biotin lambda DNA was purified from the excess nucleotides and enzyme using the Qiagen PCR Purification Kit.

For the parallel force extension measurements, we made the half-length lambda DNA functionalized with digoxigenin and biotin. First, the biotin-labelled full-lambda DNA construct was cut near the middle using the Xbal restriction enzyme (R0145S, New England Biolab). The resulting overhangs were functionalized with digoxigenin to produce a heterobifunctional 24-kbp construct labelled with digoxigenin on one side and biotin on the other.

### CFM sample cell

The mini-CFM sample cell was constructed using double-sided Kapton tape sandwiched between a 25 mm diameter support glass and a 19 mm diameter cover glass (3346, Gold Seal). The 0.7-mm-thick support glass was ordered from S.I. Howard Glass (D263) and two 1 mm diameter ports were drilled that served as the solution inlet and outlet. The cover and support glasses were cleaned by immersing in 100 ml a 1% (v/v) Hellmanex III solution, microwaving for 1 min, then sonicating for 30 min. Subsequently, the slides were rinsed thoroughly with Millipore water then dried with nitrogen flow. A 1 × 7 mm rectangular flow channel was cut on the double-sided Kapton tape using a cut plotter (Graphtec). To form tethers with digoxigenin-functionalized construct, we functionalized the cover glass with anti-digoxigenin using a modified version of a previously developed protocol^33^. First the cover glass was coated with a nitrocellulose solution by depositing 2 μl of amyl acetate solution with 0.2% (m/v) dissolved nitrocellulose. We then incubated the channel with phosphate buffered saline (PBS, 137 mM NaCl, 2.7 mM KCl, 10 mM phosphate buffer, pH 7.4) solution containing 100 μg ml^−1^ anti-digoxigenin (11333089001, Roche) for 15 min. The channel was then washed and further incubated with a surface passivation solution (10 mg ml^−1^ Roche Blocking Reagent in PBS) for 1 h. After the passivation step, the channel was flushed with experimental buffer then incubated with 5 pM of construct for 15 min. At 5 pM construct concentration, the construct was limited to an average spacing of roughly 2 μm on the surface, making formation of double tethers a rare event. After tethering, the construct to the surface, the flow channel was washed with 20 μl of the experimental buffer then incubated with 15 mg ml^−1^ streptavidin beads (M-270, Invitrogen). For each experiment, beads were washed excessively with the experimental buffer before loading them to the sample cell. Before loading the sample cell to the mini-CFM, the solution inlet and outlet ports were sealed with vacuum grease. The tris experimental buffer in Fig. 2 consisted of 10 mM Tris, 30 mM NaCl at pH 7.5 with or without 10 mM MgCl_2_.

For the overstretching experiment, the surface tethering was strengthened by replacing digoxigenin–anti-digoxigenin with biotin–streptavidin—in other words, biotin–streptavidin interactions were used to anchor both ends of each tether. The nitrocellulose surface was functionalized by incubating it with 1 mg ml^−1^ streptavidin in PBS solution that contained 1 mg ml^−1^ of Roche Blocking Reagent for 12 h, followed by incubation with passivation solution (10 mg ml^−1^ Roche Blocking Reagent in PBS buffer) for 1 h. The channel was flushed with PBS and incubated with 5 pM of dual-biotin λ-DNA for 15 min before loading in the streptavidin-coated beads. Under such conditions, the density of streptavidin on the surface was sparse enough that only one end of the biotin-labelled λ-DNA bound to the surface, leaving the other biotinylated end free to bind to the streptavidin-coated bead.

### Swinging bucket angle measurement

To measure the angle of the swinging bucket relatives to the axis of rotation, we attached an oil-based marker to the bottom of the bucket, which marks the height of the bucket on the wall as the centrifuge spins (Supplementary Fig. 2d). The uncertainty of the angle measurement was based on the distance measurement error estimate of 1 mm. At the rotational speed of 300 r.p.m. the bucket swings out to an angle of (81.4±0.8)°. At a much higher speed of 1,800 r.p.m. the angle increases by 2.3%. Further increase of the rotational speed does not increase the angle beyond the error of the measurement.

### Data analysis

The loop opening signature was identified by tracking the beads' *x* and *y* positions. As the angle of the centrifuge swing bucket was not 90° with respect to the axis of rotation, a component of the centrifugal force was directed in the *x–y* plane (Supplementary Fig. 5). As the rotational speed of the CFM increased, the looped DNA tethers were continuously extended. The opening of the loop was identified as a discontinuous change in extension. Each movie contained ∼1,000 beads. To track these beads, they were first identified using the Matlab function imfindcircles. A template image for each bead was stored. To identify the bead in the subsequent frame, the template image was scanned in the *x–y* plane to find the position of maximum correlation. First the image was scanned in the *x* direction over a 25 pixel search region centred on the bead position from the previous frame. A 2nd order parabola was then fit to the correlation coefficient as a function of position. The position of maximum correlation was identified as the new bead position. The template image was than centred on the new *x* position, and the same procedure was done in the *y* direction. During the course of each experiment, there was some drift in the *x–y* plane. This was corrected for by taking the median change in *x* and *y* for all beads being tracked from frame to frame. We found this drift correction to be sufficient for identifying the looped to unlooped transition. Transitions were identified by filtering out all bead trajectories except those that contained a discontinuous change in extension of both the correct magnitude and direction (Supplementary Fig. 5b,c). This removed false transitions that may have resulted from non-specific interactions or the formation of multiple bonds between the bead and the surface. Following this automated filtering procedure, the transition events were visually inspected, in random order, to reject any remaining erroneous transitions, which may have occurred due to particle tracking artifacts that manifest as discontinuous changes in position (that is, particle mislabeling, overlapping beads and so on).

For DNA unzipping experiments, the number of transitions as a function of time was converted to the number of transitions as a function of force as follows: The rotational speed of the centrifuge was recorded during each movie using WinMess software (provided by Thermo Fisher Scientific, R&D) which enables communication and control with the computer. The rotational speed was then converted to force (*F*) as a function of time using the following equation:


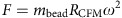


where *R*_CFM_ and *ω* are the rotational radius and speed, respectively. To determine the effective mass of the beads (*m*_bead_) in solution, we measured the Invitrogen M-270 bead density through sink-float analysis using aqueous sodium polytungstate solution (71913, Sigma-Aldrich) and obtained a density of 1.61±0.02 g cm^−3^ that confirmed the manufacturer's (Life Technologies) reported value of 1.6 g cm^−3^. The manufacturer also provided the bead diameter of our specific lot giving a mean of 2.80 μm with <1.6% CV (lot #144315600). The density of the silica beads used in the DNA force extension and overstretching experiment was measured similarly using the sodium polytungstate solution, yielding a density of 1.50±0.03 g cm^−3^. The diameter of the silica beads was measured using transmission electron microscopy, yielding an average and s.d. of 4.27±0.16 μm.

### Tethered particle motion

We carried out tethered particle motion analysis for our tethered beads prior to the rupture-force measurement. We analysed the lateral fluctuations of each bead, calculating the root mean square of the drift-subtracted displacement and the symmetry ratio, following previously established methods^34^. Here the symmetry ratio was calculated as the square root of the ratio between the minimum and maximum eigen values of the covariance matrix for the in-plane displacement. The in-plane position of each bead was recorded for 10 s at an acquisition rate of 10 Hz.

### Repeated cycles of rupture-force measurement

Multiple rupture events were collected for each molecule in a set by repeatedly spinning the same sample multiple times. For the data presented in Fig. 3, the sample was spun up with an effective force-loading rate of 1 pN s^−1^ to a maximum force of 22 pN. The speed was then ramped down to 0 r.p.m. for ∼1 h to allow rebinding between each pair of molecules. Beads were identified from cycle to cycle by their positions relative to a set of fiducial beads, which were common to each cycle. For the measurement of the two populations of molecules, we made two different DNA nanoswitch unzipping constructs, one with 48% GC content (5′-CACGAATTCTCTGCCTCCCTTTTAACCCTAG-3′) and one with 31% GC content (5′-CTCAAATATCAAACCCTCAATCAATATCT-3′).

## Additional information

**How to cite this article:** Yang, D. *et al*. Multiplexed single-molecule force spectroscopy using a centrifuge. *Nat. Commun.* 7:11026 doi: 10.1038/ncomms11026 (2016).

## Supplementary Material

Supplementary Information Supplementary Figures 1-6, Supplementary Table 1, Supplementary Notes 1-3 and Supplementary References

Supplementary Data 1 Solidworks file for the CFM Assembly

Supplementary Data 2 Solidworks file for CFM Holder 1

Supplementary Data 3 Solidworks file for CFM Holder 2
