# Supplementary material for: Multiplexed single-molecule force spectroscopy using a centrifuge
Source: Nat Commun. 2016 Mar 17;7:11026. doi: 10.1038/ncomms11026 (PMC4800429; doi:10.1038/ncomms11026)
Supplement: Supplementary Information — Supplementary Figures 1-6, Supplementary Table 1, Supplementary Notes 1-3 and Supplementary References [file ncomms11026-s1.pdf]

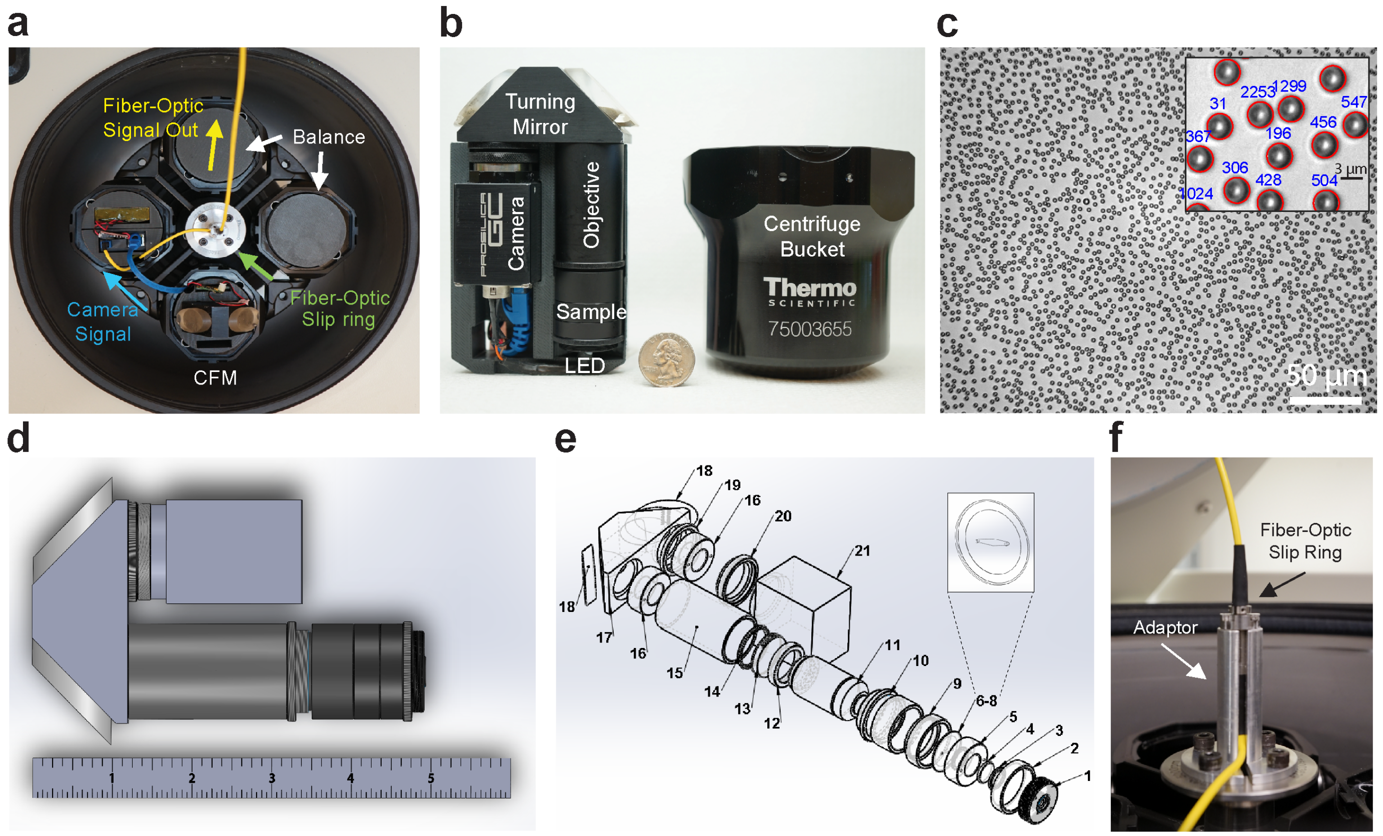

Supplementary Figure 1 | Detailed design of the benchtop CFM. (**a**) Photograph of the benchtop CFM module inside of a centrifuge. (**b**) The benchtop CFM module next to a typical centrifuge bucket. The LED, sample holder, and objective are housed within the lens tubes that are mounted to the turning mirror and camera. (**c**) Typical field of view during a CFM force spectroscopy experiment showing thousands of surface-tethered beads that can be monitored in parallel. (**d**) Assembled model of the CFM. The full length of the CFM is less than 5 inches long and it is compact enough to fit into a conventional benchtop centrifuge bucket. (**e**) Exploded view of the CFM. The majority of the parts can be purchased and threaded for direct assembly without the need for user alignment. **Supplementary Table 1** contains a list of parts with detailed purchasing information. The insert (Part 6-8) is the illustration of the sample coverslip. (**f**) Installation of the fiber-optic rotary joint onto the benchtop centrifuge. The computer-aided design (CAD) drawings of the CFM assembly and customized parts are included as supplemental material.

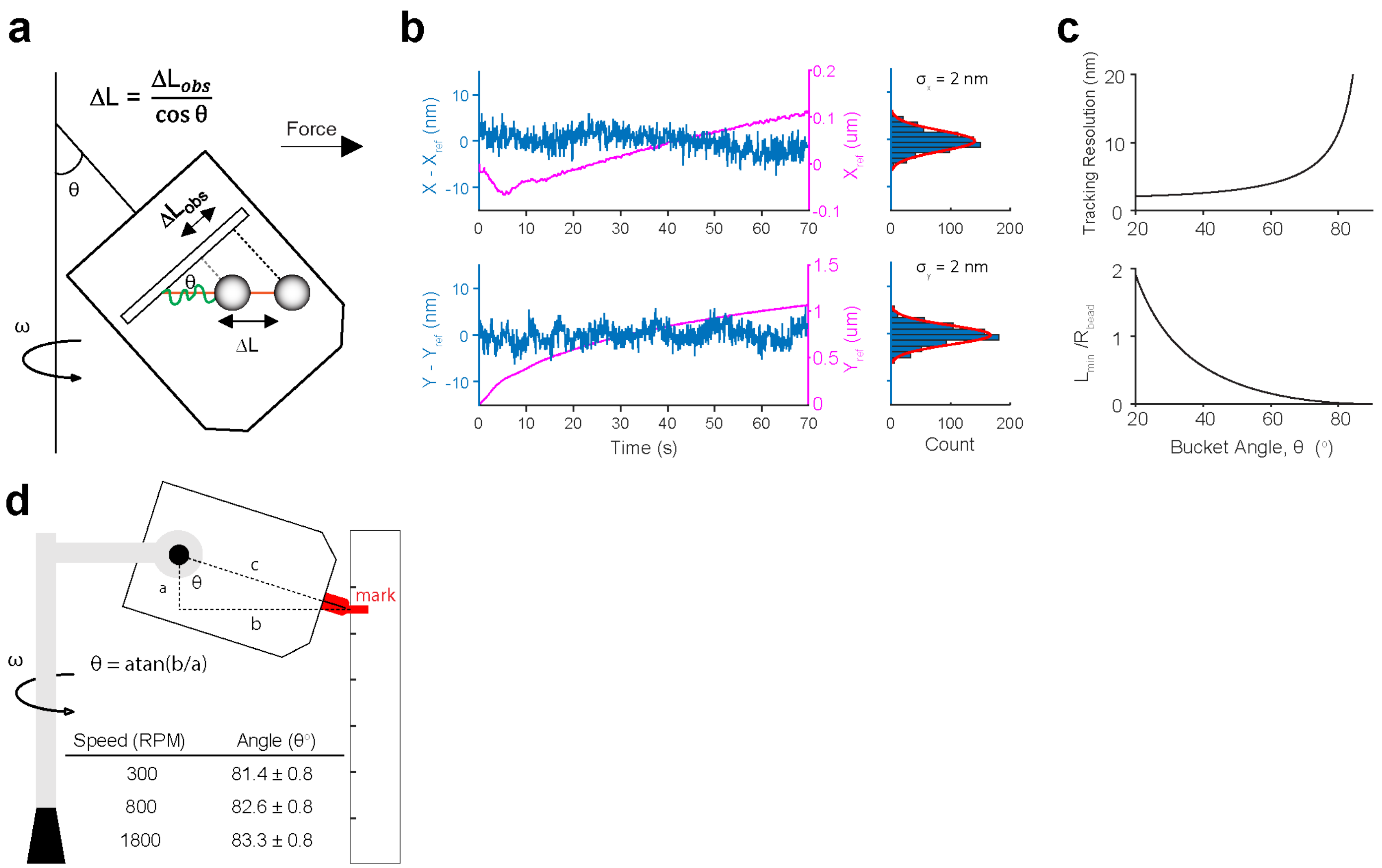

Supplementary Figure 2 | Characterization of bead x-y position tracking. (**a**) Illustration of tether extension measurements obtained using XY projection when the direction of force and imaging axis are intentionally misaligned. Changes in the length of molecular tethers can be calculated using the observed lateral shift in the position of a tethered bead (ΔL_{obs}), and the bucket angle (θ). (**b**) Drift-corrected x and y position of 5 um silica stuck bead as a function of time recorded in the centrifuge spinning at 2,000 RPM is shown in blue and the right panel shows the histogram of the position with normal distribution fits. Standard deviations of the fits for x and y positions are both ~2 nm. The drift in x and y position as a function of time, based on the average position of 12 immobile reference beads, is shown in magenta. While the standard deviation listed here represents the particle-tracking resolution, we note that the overall accuracy with which tether lengths can be measured also depends on the intrinsic thermal fluctuations of these beads as previously described[2] (**c**) Top panel shows Tracking resolution of tether extension calculated as a function of the bucket angle as illustrated in (**a**). The bottom panel shows the Minimum tethered length (L_{min}), scaled by the bead radius (R_{bead}), required to measure tether extension from lateral displacement, calculated here as a function of the bucket angle. (**d**) Illustration of the angle measurement. The angle is determined from the height of the swinging bucket while the centrifuge spins. The inserted table shows the angle at different rotational speeds.

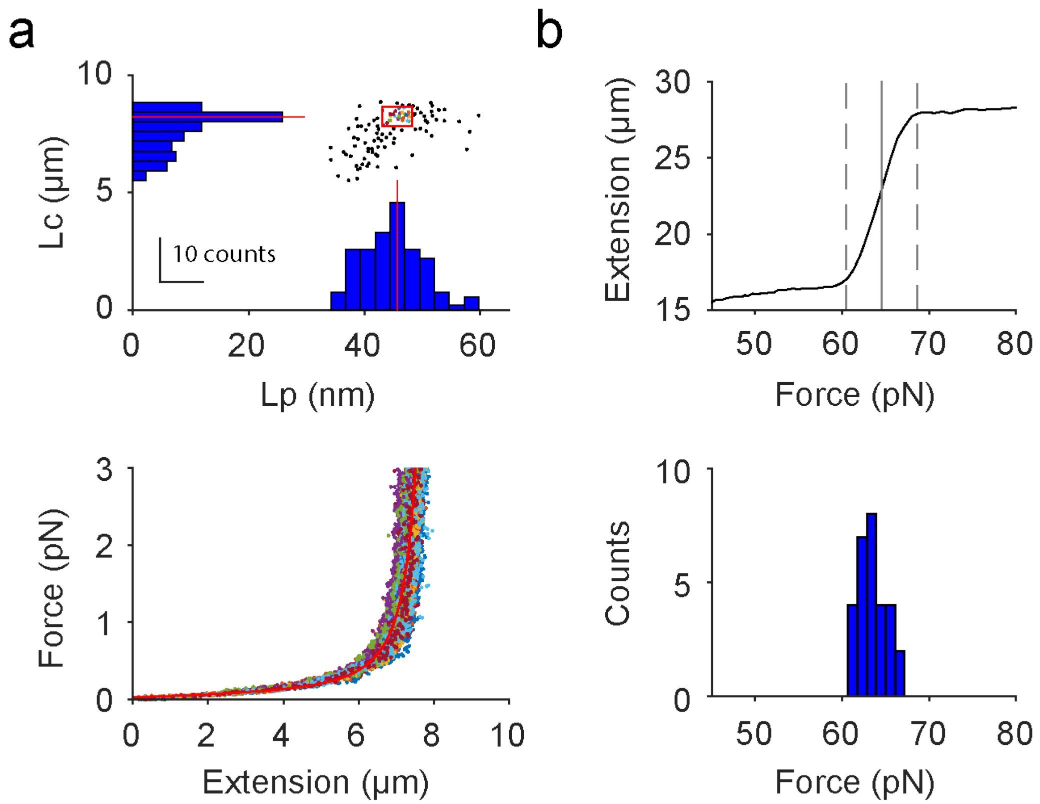

Supplementary Figure 3 | Parallel DNA force-extension and overstretching measurements made with the CFM. (**a**) Force-extension data of half lambda DNA (24 kbp) obtained from a single sample with 113 DNA tethers using a centrifuge bucket constrained to a 20° angle (see Supplementary Fig. 2a for detail). The top panel shows a scatter plot of the persistence length and contour length obtained from fits to the worm-like chain model performed for each tether (n=113)., Histograms projecting the persistence length and contour length of the model onto the x- and y-axis, respectively, are shown with red lines indicating expected values. The bottom panel shows the force-extension curves of single-tethered DNA data filtered by persistence length and contour length (n=30). Ranges were selected from the peaks of the histograms (one bin-width on either side), yielding filtering ranges of 43-48 nm and 7.8-8.6 µm for the persistence length and contour length, respectively. The overlayed red curve represents the expected force-extension curve. (**b**) Multiplexed DNA overstretching measured with the benchtop CFM. The top panel shows a representative force-extension curve near the overstretching transition. The overstretching force was extracted as the half-way-point of the two overstretching transition forces and is shown as a vertical grey line between the dashed vertical lines. The bottom panel shows a histogram of the overstretching force measured from a single sample (n=29) with an average and standard deviation of 63.5 ± 1.7 pN.

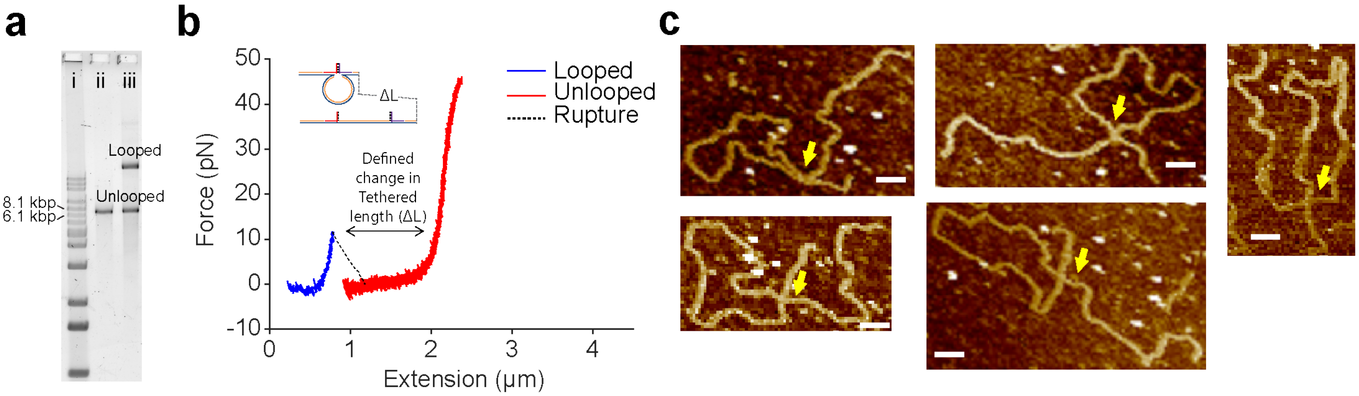

Supplementary Figure 4 | Verification of DNA Unzipping Nanoswitch Construct. (**a**) Gel electrophoresis of the DNA nanoswitch showing loop formation. Lane i is the 1 kbp extension ladder (Invitrogen, 10511-012), lane ii is a linear construct without the two complementary oligos that close the loop, and lane iii is the nanoswitch construct with the two complementary oligos that can form the looped nanoswitch. The looped DNA migrates more slowly in the gel than the linear construct, resulting in a discrete band with a higher apparent molecular weight as previously observed[3,4]. (**b**) Force-extension curve of the DNA unzipping nanoswitch construct measured using optical tweezers. The blue curve corresponds to the looped construct. When forces above ~12 pN were applied, the 29 bp dsDNA that formed the loop unzipped, causing the tether length to increase to the full length of the M13 dsDNA tether. (**c**) AFM images of the construct. The yellow arrow indicates the putative location of the hybridized DNA zipper. The length of the scale bar is 100 nm.

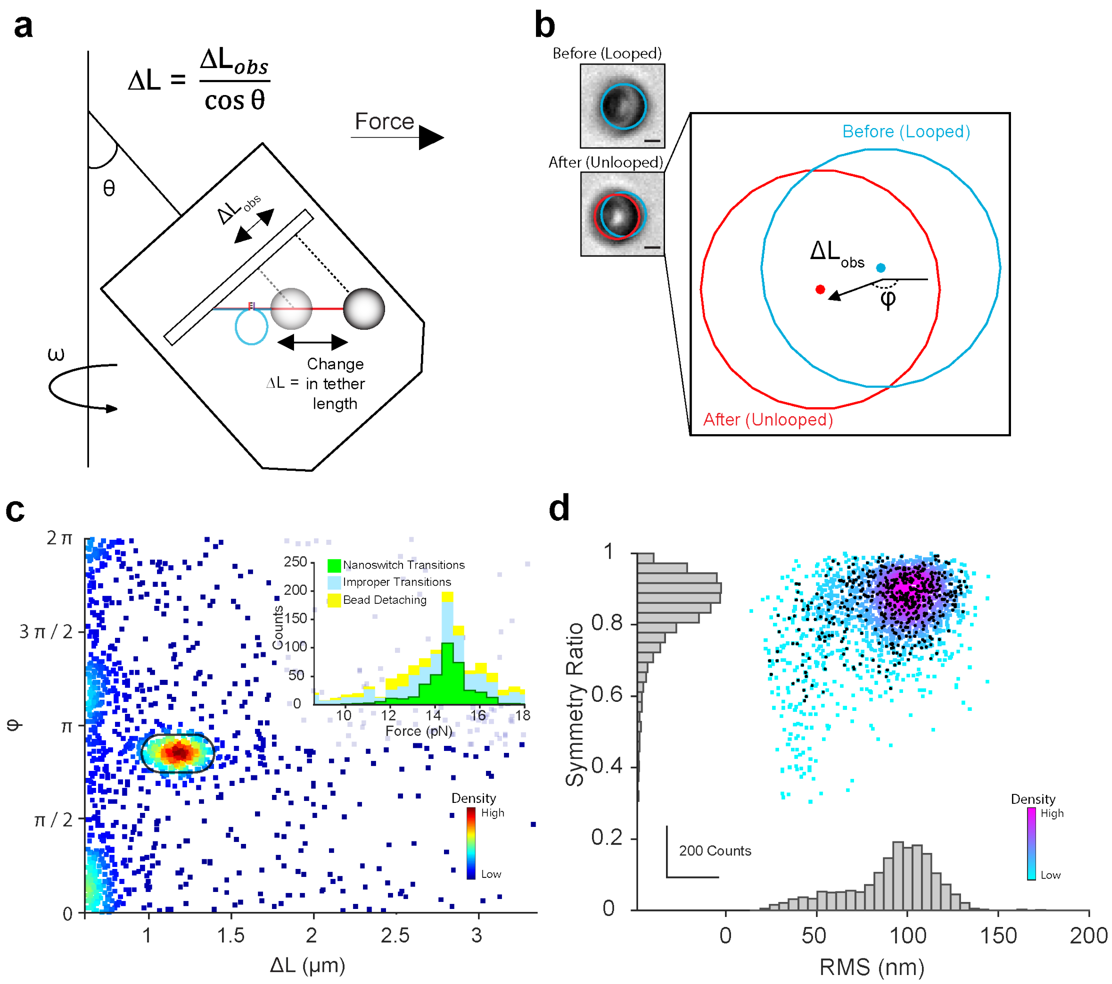

Supplementary Figure 5 | DNA Nanoswitches as Molecular Signatures. (**a**) Experimental geometry. The centrifuge bucket is at angle, θ, therefore the centrifugal force has components both perpendicular and parallel to the sample surface (x-y plane). When the bond is ruptured, the DNA Nanoswitch goes from looped (blue) to unlooped (red) experiencing a change in length ΔL. This is identified by measuring the projected change in length in the xy plane $\Delta L_{obs} = \Delta L \cos\theta$. The orientation of the miniCFM in the bucket sets the direction of ΔL_{obs}. (**b**) Images of a bead before (above), and after (below) a loop opening transition. A bead which is tethered to the surface with a single DNA nanoswitch will undergo a discontinuous change in position with a well-defined length $\Delta L_{obs,}$ and direction φ. (**c**) A scatter plot of all contour length changes detected for all directions. Color represents data density. Only transitions within the boxed region are accepted as nanoswitch transitions. Inset, a histogram of transition forces for three different types of transitions: beads which leave the surface (yellow), beads which display discontinuous transition with (green) and without (light-blue) correct direction and magnitude. (**d**) A scatter plot of the symmetry ratio and root mean square displacement based on the lateral fluctuations of all tracked beads, with color representing data density. The overlayed black data points are for tethered beads that undergo validated nanoswitch transitions.

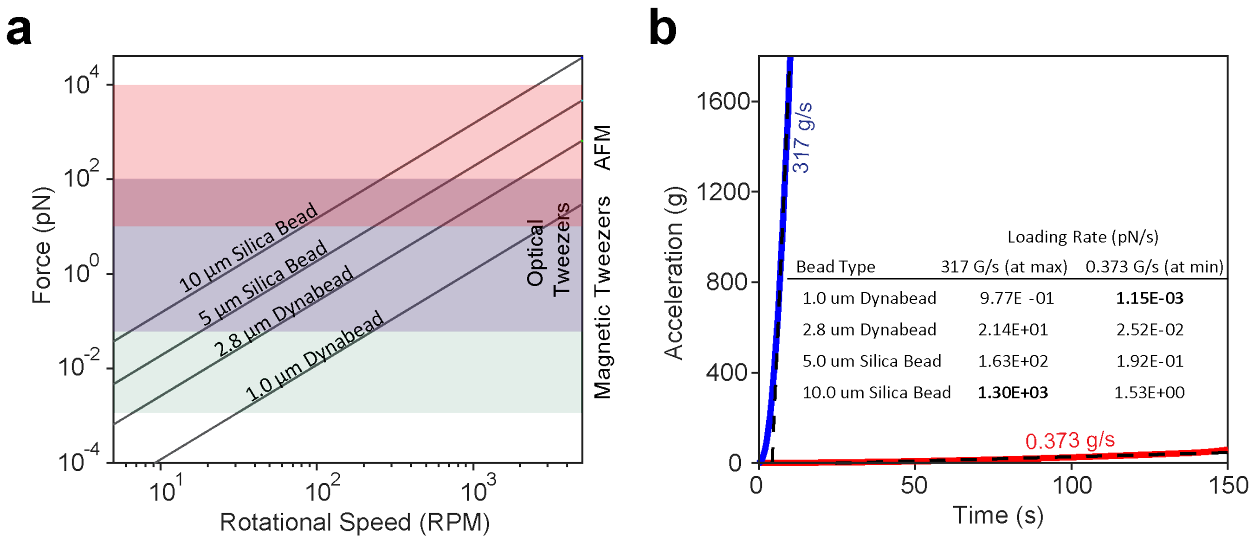

Supplementary Figure 6 | Force and loading rate range of the benchtop CFM. (**a**) Force as a function of rotational speed calculated using four different types of beads (1 and 2.8 um Dynabeads, and 5 and 10 um silica beads) for the benchtop CFM. Using this set of beads, the CFM is capable of applying a force range that spans eight orders of magnitude (10^{-4} to 10^{4} pN). (**b**) The fastest and slowest force-loading rates of the benchtop CFM measured using the Thermo Scientific Heraeus X1R Centrifuge. The fastest ramping rate of 317 g/s, shown in blue, can correspond to a 1,300 pN/s loading rate using a large 10 um silica bead. The slowest ramping rate of 0.373 g/s, show in red, can corresponds to a 1.15 fN/s loading rate using a small 1.0 um Dynabead (Invitrogen).

TEM NO.	VENDOR	PART NUMBER	DESCRIPTION	QTY.	Price
1	Thorlabs	S1LEDM	SM1-Threaded Mount for LED	1	27.00
2	Thorlabs	SM1T1	SM1 (1.035"-40) Coupler	1	9.50
3	Thorlabs	SM05RR	Retaining Ring for diffuser	1	4.00
4	Thorlabs	DG05-220	Light Source Diffuser, Ø1/2" N-BK7 Ground Glass	1	12.10
5	Thorlabs	SM1A6T	Diffusr and sample cell mount	1	19.75
6	SI Howard Glass Co	D265	Ø 25 mm, 0.7 mm Thick	1	-
7	Kapton Tape	PPTDE-1	Double Sided tape for Sample Cell Assembly	1	-
8	Electron Microscopy Sciences	63782-01	Gold Seal, #1 19 mm coverglass	1	-
9	Thorlabs	SM1L03	Sample Holder, SM1 Lens Tube, 0.3" Thread Depth	1	12.16
10	Thorlabs	SM1V05	Focusing Ø1" SM1 Lens Tube	1	29.60
11	Edmund Optics	#86-815	40X Olympus Plan Achromat Objective, 0.65 NA, 0.6 mm WD	1	575.00
12	Thorlabs	SM1A3	Objective Adapter with External SM1 Threads and Internal RMS Threads	1	16.75
13	Thorlabs	AC254-100	Tube Len, f=100.0 mm, Ø1" Achromatic Doublet, ARC: 400-700 nm	1	73.40
14	Thorlabs	SM1RR	Tube Lense SM1 Retaining Ring	1	4.50
15	Thorlabs	SM1M20	Objective SM1 Lens Tube Without External Threads, 2" Long	1	15.50
16	Thorlabs	SM1A6T	Adapter with External SM1 Threads and Internal SM05 Threads, 0.40" Thick	2	39.50
17	-	-	Custom made turning block, aluminum	1	-
18	Thorlabs	PFE10-P01	Turning Mirror, 1" Silver Elliptical Mirror, 450 nm - 20 μm	2	123.60
19	Thorlabs	SM1NT	Camera SM1 (1.035"-40) Locking Ring, Ø1.25" Outer Diameter	1	6.50
20	Thorlabs	SM1A9	Camera Adapter with External C- Mount Threads and Internal SM1 Threads	1	18.75
21	Allied Vision Technologies	Prosilica GC2450	Sony ICX625 CCD sensor, 2448 x 2050 resolution, 15 fps, 12 bit	1	3000.00
22	IMC Network	855-10734	MiniMc-Gigabit Twisted Pair to Fiber Media Converter	1	260.00
23	PrinceTel	MJX	Fiber Optic Rotary Joint	1	750.00
				**Total**	4997.61

Supplementary Table 1 | Parts list of the benchtop CFM including vendor information. The item numbers listed here correspond to the item numbers in Supplementary Fig. Y. The parts highlighted in the table are discussed in detail in **Supplementary Note 3**, together with suggestions for more economical substitutes.

Supplementary Note 1.

Based on the geometry as illustrated in (**Supplementary Fig. 2a**), extension of the molecular tether can be measured by tracking the tethered microsphere's motion parallel to the coverslip. Specifically, changes in tether extension will appear as a lateral displacement of the bead (ΔL_{obs}). The actual changes in extension (ΔL) in the direction parallel to the force can be calculated based on the angle of the bucket (θ) as follows:

$$\Delta L = \frac{\Delta L_{obs}}{\cos(\theta)}$$

The minimum tether length that can be measured in this way depends on both the bead size used in the experiment and the angle of the centrifuge bucket. The bead can make direct contact with the cover glass surface if the tethered length is not long enough for the bead to be pulled away from the surface. The minimum tether length (L_{min}) as a function of bead radius (R_{bead}) and bucket angle (θ) is given by:

$$L_{min} = R_{bead}\left(\frac{1}{\sin(\theta)} - 1\right)$$

The tracking resolution of tether extension (ΔL) and values of L_{min} in units of bead radius (R_{bead}) are calculated for bucket angles between 20° to 85° as shown in **Supplementary Fig. 2c**.

Supplementary Note 2.

The unzipping force of the 29 bp DNA duplex measured here increases as the temperature decreases in reasonable quantitative agreement with simple thermodynamic theoretical predictions. Interestingly, the theoretical prediction slightly underestimates the unzipping force at low temperature (**Fig. 2c**). Previously, Danilowicz et al. measured the temperature-dependent unzipping force of a 1.5 kbp long DNA, and noted an unzipping force at 15 ^{o}C of 36 pN that was 3 times larger than the predicted value of 12 pN[1]. This surprising observation was attributed to a DNA conformational change that occurs at low temperature. This underestimate in the unzipping force that results from the thermodynamic analysis was not as pronounced in our much shorter DNA sequence, suggesting that the low temperature DNA conformational change proposed to explain the earlier results may depend strongly on the length of the unzipped DNA.

Supplementary Note 3.

The benchtop CFM bill of materials is summarized in **Supplementary Table 1** and the exploded view drawing of the assembly is shown in **Supplementary Fig. 1e**. The majority of the opto-mechanical components can be purchased from Thorlabs. Comparable components such as the lenses, objective and mirror can be purchased from other vendors as well. The objective (Item 11), camera (Item 21), and fiber optic rotary joint (Item 23) account for most of cost of this CFM design. The camera used here is equipped with a 5 Megapixel CCD sensor with a maximum frame rate of 15 fps at full resolution. Depending on the application, alternative cameras with different resolutions and acquisition rates could be used to reduce the total cost of construction—for example, the Basler Ace CMOS camera at 15 Megapixels is under $700 and a 2 Megapixel version is under $400 (Edmund Optics, Inc.), which would reduce the total price to about $2,500. The media converter (Item 22) that converts the twisted wire Ethernet connection to an optical signal could be omitted if a camera with a 10 GigE optical fiber output is used. Data output using a compact wireless router is an alternative

approach, but may result in a slower acquisition rate. Additional customized parts such as the turning mirror housing can be replaced with a Thorlabs compact cage cube system, but for added stability of the imaging path, we recommend a solid aluminum construction. Moreover, larger floor model centrifuges with 1 Liter buckets can provide sufficient bucket depth that the turning mirror is no longer needed. The CAD drawing of the solid aluminum turning mirror housing and the full CAD assembly of the CFM show in **Supplementary Fig. 1** is available in the **Supplemental Files**.

SUPPLEMENTARY REFERENCES

1 Danilowicz, C. *et al.* Measurement of the phase diagram of DNA unzipping in the temperature-force plane. *Phys Rev Lett* **93**, doi:Doi 10.1103/Physrevlett.93.078101 (2004).

2 De Vlaminck, I. & Dekker, C. Recent advances in magnetic tweezers. *Annual review of biophysics* **41**, 453-472 (2012).

3 Halvorsen, K., Schaak, D. & Wong, W. P. Nanoengineering a single-molecule mechanical switch using DNA self-assembly. *Nanotechnology* **22**, doi:Doi 10.1088/0957-4484/22/49/494005 (2011).

4 Koussa, M. A., Halvorsen, K., Ward, A. & Wong, W. P. DNA nanoswitches: a quantitative platform for gel-based biomolecular interaction analysis. *Nat Methods* **12**, 123-U148, doi:Doi 10.1038/Nmeth.3209 (2015).
